# Tandem Repeat Modification during Double-Strand Break Repair Induced by an Engineered TAL Effector Nuclease in Zebrafish Genome

**DOI:** 10.1371/journal.pone.0084176

**Published:** 2013-12-26

**Authors:** Wanxu Huang, Jianbo Zheng, Ying He, Chen Luo

**Affiliations:** College of Life Sciences, Zhejiang University, Hangzhou, Zhejiang, China; Imperial College London, United Kingdom

## Abstract

Tandem repeats (TRs) are abundant and widely distributed in eukaryotic genomes. TRs are thought to have various functions in gene transcription, DNA methylation, nucleosome position and chromatin organization. Variation of repeat units in the genome is observed in association with a number of diseases, such as Fragile X Syndrome, Huntington's disease and Friedreich's ataxia. However, the underlying mechanisms involved are poorly understood, largely owing to the technical limitations in modification of TRs at definite sites in the genome *in vivo*. Transcription activator-like effector nucleases (TALENs) are widely used in recent years in gene targeting for their specific binding to target sequences when engineered *in vitro*. Here, we show that the repair of a double-strand break (DSB) induced by TALENs adjacent to a TR can produce serial types of mutations in the TR region. Sequencing analysis revealed that there are three types of mutations induced by the DSB repair, including indels only within the TR region or within the flanking TALEN target region or simutaneously within both regions. Therefore, desired TR mutant types can be conveniently obtained by using engineered TALENs. These results demonstrate that TALENs can serve as a convenient tool for modifying TRs in the genome in studying the functions of TRs.

## Introduction

Even years before the nucleotide sequencing methods were developed, a great mass of repeated sequences were detected in vertebrate genomes by denaturation-renaturation experiments [1]. Nowadays, whole-genome sequencing of various model organisms demonstrated that repetitive sequences are abundant and widely distributed in eukaryotic genomes. Typically, repeat sequences can be classified into two families: dispersed repeats and tandem repeats. Dispersed repeats are so called because of their interspersed distribution throughout the genome, such as transposons and gene paralogues; while repeat units in tandem repeated sequences are located next to other (i.e., in tandem). Each of the two families can be divided into subfamilies according to their sequence and distribution characteristics. When genomic DNA being separated by density-gradient centrifugation, tandem repeats (TRs) will be identified as satellite bands, so TRs are also named satellite DNA, among which, repeats with relatively large units (9 nucleotides long and above) are generally called minisatellites [2], and repeats with smaller units (from 1–8 nucleotides) are defined as microsatellites, also called short tandem repeats (STRs) or simple sequence repeats (SSRs) [3].

TRs can distribute everywhere in the genome, including gene bodies, untranslated regions (UTRs) and promoters [4]. Although tandem repeat sequences were historically regarded as nonfunctional junk or selfish DNA [5], [6], they have attracted great attention since early 1990s when several significant diseases were reported due to tandem repeats disorder, such as Fragile X Syndrome [7], [8], Huntington’s disease [9] and Friedreich's ataxia [10], and to date, more than 20 diseases identified are in association with abnormity of tandem repeats [11]. Thus, TRs have been suggested as a third category of genetic variation, besides of single nucleotide polymorphisms and copy number variations [12]. Apart from their roles in diseases, TRs are also thought to have various functions in gene transcription, DNA methylation, nucleosome positioning and chromatin organization [13]–[15]. However, the underlying mechanisms are poorly understood, largely owing to the technical limitations in modification of TRs at definite sites in the genome *in vivo*.

Transcription activator-like (TAL) effectors are sequence-specific DNA binding domain proteins identified from plant pathogenic bacteria *Xanthomonas* in recent years [16], [17]. Synthetic genes encoding TAL effector nucleases (TALENs) can be constructed *in vitro* by fusing TAL effector with *Fok* I nuclease, so that TALENs can recognize specific DNA sequences (by TAL effector) followed by creating a double strand break (DBS) in the target site (by *Fok* I nuclease). In living cells, DSBs can be repaired in two pathways [18], [19]: the non-homologous end-joining (NHEJ) and homologous recombination (HR). In the former pathway, the broken sites are simply rejoined in an error-prone fashion and hence usually leading to small insertions or deletions (indels) at the DSB sites; while in the later pathway, the DNA surrounding the DSB site is replaced with a homologous template sequence. Here, we show that TALENs can serve as a convenient tool for modifying the TRs in the genome in studying the functions of TRs. This technique might be also a potential therapeutic approach for aforementioned genetic disorders resulted from TR instability.

## Materials and Methods

### Animals and Ethics Statement

Zebrafish (*Danio rerio*) used in our experiments is long-fin strain. Bisexual diploid goldfish (*C. auratus*) and unisexual polyploid goldfish (*C. auratus* pengze) were purchased from nearby farms and maintained in our laboratory in the breeding season. Artificial spawning and fertilization were performed as previously reported [20]. This study was approved by the Ethics Committee of Laboratory Animal Center of Zhejiang University (Zju201306-1-11-060).

### Obtaining the upstream sequence of *ntl* in zebrafish and goldfish

The upstream sequence of zebrafish *no tail* (*ntl*), a decisive developmental regulatory gene, was obtained from the nucleotide database in EMBL. The upstream sequence of goldfish *ntl* was obtained after four rounds of genome walker using GenomeWalker™ Universal Kit (CloneTech, USA). The gene-specific primers (GSPs) used are listed in Table S1 (in File S1). Among which, GSPs for the third round of genome walker (GSP5 and GSP6) were designed according to the published sequence (GenBank accession NO. EU549781), and GSPs for the fourth round of genome walker (GSP7 and GSP8) were designed according to the result of the third round. Then, the totally four rounds of genome walker products were spliced and the entire fragment was validated by PCR with gene-specific primer pair (GF-ntl-promt-seq-S/AS) and sequence analysis.

### TALEN targets design

A (TG)_n_ repeat sequence far upstream region of zebrafish *ntl* was selected as a target for TALENs. A web-based tool called TAL Effector-Nucleotide Targeter 2.0 (TALE-NT 2.0; https://tale-nt.cac.cornell.edu) [21] was used to design TALEN targets. Since *Fok* I nuclease functions as a dimer when used to make double-strand breaks and the length of the spacer can affect the specificity of TALENs, TALENs were designed in pairs that bind opposing DNA target sites separated by a spacer, and the length of the spacer can affect the specificity of the TALRNs The range of spacer length used for searching potential targets was between 12-18bp, within the suggested region of optimal activity for TALENs [22]. The lengths of the repeat arrays were from 15 to 21 units. The TALEN target sequences were chosen after a T and ended with a T.

### Construction of engineered TALENs

The engineered TAL effector repeats arrays were assembled according to the designed targets, and we adopted the “unit assembly” method described by Huang et al. [23]. The units of repeats arrays were supplied by the manufacture (CWBIO, Beijing), and the procedure of assembly is just as reported formerly [23]. To construct engineered TALENs, the assembled TAL effector repeats arrays were double digested from their original vectors by *Spe* I and *Nhe* I (TaKaRa, Japan) and then cloned into pCS2-*Fok* I plasmids (CWBIO, Beijing).

### Efficiency evaluation of the TALEN pairs

The efficiencies of the TALEN pairs in inducing DBS and single strand annealing (SSA) recombination were evaluated *in vitro* by Luciferase SSA recombination assay [23], [24]. The luciferase SSA reporter (pSSA-luciferase) is composed of a CMV promoter and two homologous luciferase coding fragment separated by a stop codon and an inserted TALEN target sequence. Primers used for cloning TALEN target sequences into pSSA-luciferase are listed in Table S2 (in File S1). 100 ng of each TALEN pair were co-transfected with 50ng corresponding pSSA-luciferase into HEK293T cells (CWBIO, Beijing) in 24-well plates using TurboFect™ *in vitro* Transfection Reagent (Fermentas, Canada). 10 ng of Renilla luciferase driven by the *β–actin* promoter in co-transfection with 100 ng of corresponding TALEN pair was used as the toxic reference to judge the toxicity of designed TALEN pairs. For each sample, the test and control reactions were run in triplicate. The cells were harvested 24 hours after transfection and lysed using Luciferase Cell Lysis Buffer (NEB, USA). The relative luciferase activity was then detected by Dual-Luciferase Reporter® (DLR™) Assay System (Promega, USA) and measured by SpectraMax L Luminescence Microplate Reader (Molecular Devices, USA). The specific value of average firefly luciferase readout/average renilla luciferase readout (F/R) was calculated. The efficiency index was obtained by comparing the F/R value of TALEN with that of control.

### Transcription of TALENs *in vitro* and microinjection

The constructed pCS2-TALE-*Fok* I vectors were linearized with *Not* I (TaKaRa, Japan) as templates, from which the capped mRNAs of TALEN pair were transcribed using mMESSAGE mMACHINE Sp6 Kit (Ambion, U.S.A.). Capped mRNAs of TALEN pair were coinjected into zebrafish embryos at the 1-cell stage.

### Mutants screening in TALENs injected zebrafish embryos

TALEN-injected zebrafish embryos were maintained in 0.1× Hank’s solution at 28.5°C. The survival rate was accounted at 1 day post fertilization (dpf). Genomic DNA from single 4 dpf embryos was extracted following our previously reported procedure [20]. A ∼470bp DNA fragment (in wild type) encompassing the TALEN target site and the TR region was amplified by PCR using the primers as follows: 5'-TCCTGTTCAATGTGTTTTATCAGTATGC-3' (forward) and 5'-CTTAATTTCTTCATGTTGTTCTAATGCAA-3' (reverse). PCR products were run on the agarose gel and then validated by sequencing.

## Results

### The (TG)_n_ at the upstream region of zebrafish *ntl* is suitable for examining the effect of TALENs on TR modification

To examine the effect of TALENs on TR modification, the length of the target TRs should be long enough to induce indels and the adjacent sequence should have suitable binding sites to design TALEN pairs. Moreover, it is better that artificial length change of the target TRs would not elicit lethal effect on the embryo.

**Figure 1:**
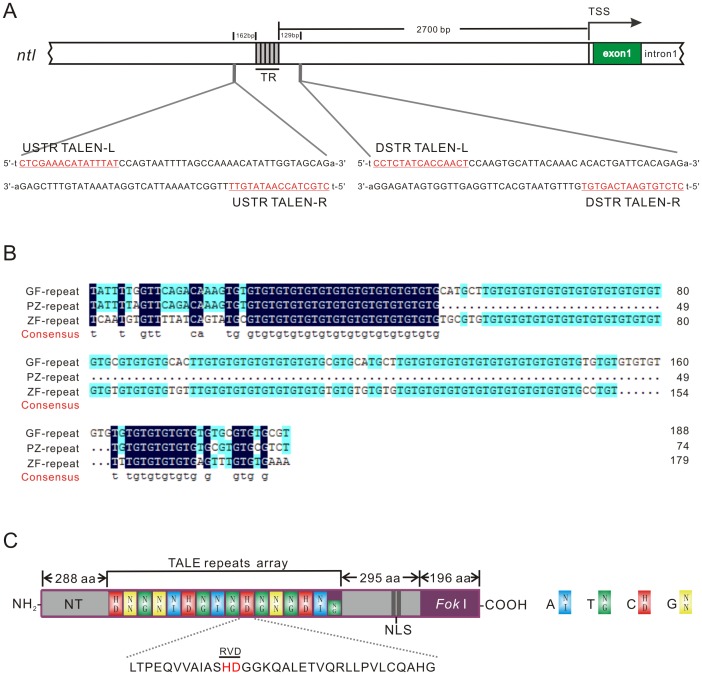
A (TG)_n_ sequence in the upstream region of *ntl* and TALEN targets design. (A) Position of a (TG)_n_ sequence in the upstream region of zebrafish *ntl* and two target sites for designing TALENs. The arrow indicates the transcription start site (TSS) of *ntl*. The (TG)_n_ region is showed in grey adjacent boxes, and the two designed TALEN targets are described below, in which red letters underlined represent the binding sites of left (L) and right (R) TALENs, respectively. All the TALEN target sites were designed with a preceding T at 5' terminal (showed in lowercase). (B) Alignment of (TG)_n_ sequence at upstream region of *ntl* among zebrafish, bisexual diploid and unisexual polyploid goldfish. GF: bisexual diploid goldfish (*Carassius auratus*), PZ: unisexual polyploid goldfish (*Carassius auratus*, pengze), ZF: zebrafish. (C) Structure of TALEN fusion protein, which is composed of a N-terminal translocation domain (NT), a central DNA binding domain, and a C-terminal domain containing a nuclear location site (NLS) and followed by a *Fok* I nuclease. The TALEN DNA binding domain typically comprises a tandem array of 13–28 single repeat unit [22], each one consisting of 34 highly conserved residues, in which the residues at positions 12 and 13 are called repeat-variable di-residue (RVD). Different RVDs associate specifically with different nucleotides, with NI, NG, HD, and NN accounting for each of the four nucleotides A, T, C and G, respectively. The end of C-terminal repeat unit (showed in the short green box) generally contains only 20 amino acids and is therefore referred to as ‘half-repeat’, which includes a RVD specifically recognizing the nucleotide T.

By searching in the nucleotide database in EMBL, we identified a 140bp long imperfect tandem TG repeat sequence (represented with (TG)_n_, where n = 70) about 2.7 kbp upstream of the zebrafish *ntl* that has two candidate TALEN-binding sites (Figure 1 A). Since *ntl* is a decisive regulatory gene of development, we examined whether this TR is evolutionary conserved and evaluated if the length change of this sequence was tolerable by comparing the upstream sequence among zebrafish, bisexual diploid and unisexual polyploid goldfish. After four rounds of genome walker, the upstream sequence of *ntl* gene in the two subspecies of goldfish was obtained and aligned (Figure S1 in File S1). Sequencing analysis showed that an imperfect (TG)_n_ repeat exists upstream of *ntl* in both bisexual diploid and unisexual polyploid goldfish. The position of (TG)_n_ in both subspecies of goldfish is in accordance with that in zebrafish. Aligning comparison showed that the length of the (TG)_n_ between zebrafish and the bisexual diploid goldfish is almost equal, but remarkably shorter (about 110bp) in the unisexual polyploid goldfish (Figure 1 B). This result suggests that the (TG)_n_ is an evolutionary conservative element and the length change is tolerable. Therefore, the (TG)_n_ upstream of zebrafish *ntl* is suitable for examining whether engineered TALENs can be employed to modify the length of TRs.

### An optimal target site for TALEN pair is at the downstream of the TR

In order to screen an optimal TALEN pair for inducing a DBS and SSA recombination, two pairs of TALEN plasmids, named USTR TALEN-L/R and DSTR TALEN-L/R, were designed and constructed targeting to the adjacent upstream (162bp) and downstream (129bp) of the (TG)_n_ (Figure 1A). All the TALEN target sites were designed with a preceding T. Each TALEN pair was designed with a spacer length of 17bp and repeats arrays length of 15 or 16 units (all the last units contained only 20 amino acids and were therefore referred to as ‘half-repeats’, specifically recognizing the nucleotide T). The structure of TALEN fusion protein is described in Figure 1C.

**Figure 2:**
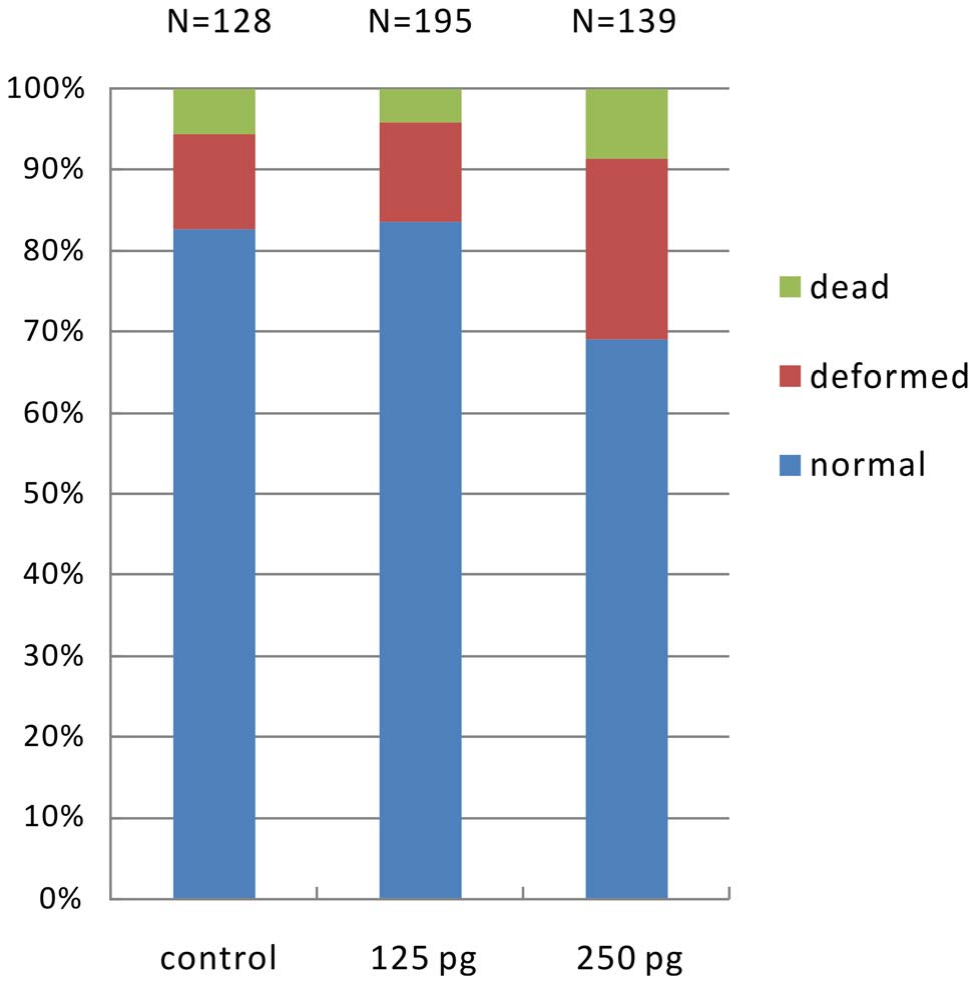
Statistics of zebrafish embryos after one day of TALENs injection at different dosage. The number of embryos scored (N) is indicated at the top and the dosage is indicated at the bottom.

To determine the efficiency of the two TALEN pairs, two firefly luciferase SSA reporters were constructed by inserting the USTR TALEN or DSTR TALEN target sequences into the cloning site (Figure 2A). Because the coding region of the firefly luciferase was engineered with two 870bp homologous arms, which were separated by a stop codon and a TALEN target sequence, no active firefly luciferase would be expressed from the reporter plasmid before co-transfection of a functional TALEN pair. The binding of a functional TALEN pair will create a DSB, which after error-prone NHEJ-mediated repair can generate an active firefly luciferase gene (Figure S2 in File S1). Thus, comparing to the firefly luciferase SSA reporter transfected cells, the gain of firefly luciferase signal in the firefly luciferase SSA reporter and the corresponding TALEN pair co-tansfected cells can be taken as an indicator of TALEN activity, while the loss of *β–actin* promoter driven Renilla luciferase signal in the toxic control can be seen as an indicator of TALEN toxicity, due to off-target cleavage usually causing apoptosis of the transfected cells [24].

Luciferase detection showed high level of Renilla luciferase signal in the URTR TALEN or DSTR TALEN pair co-tansfected control cells (Table 1), suggesting that the cell toxicity of both TALEN1 and TALRN-2 pairs is very low. As showed in Table 1, the efficiency index of DSTR TALEN pairs in inducing a DBS and SSA recombination was much higher than that of URTR TALEN, indicating that the optimal target sequence for TALEN pair is at downstream of the TRs. Therefore, DSTR TALEN pair was chosen for the further experiment.

**Table 1:** Efficiency and toxicity evaluation of the TALEN pairs *in vitro* by SSA assay.

	USTR TALEN		DSTR TALEN	
	untransfected	transfected	untransfected	transfected
Firefly Luciferase	549473.23	5032081.60	1069911.29	27217327.77
	549367.17	5040331.13	1041310.74	28502196.83
	558803.04	5038530.93	1036584.63	28666427.67
Renilla Luciferase	35837696.22	95180434.34	114368257.99	89138298.60
	37807751.30	96921199.88	111730895.80	91984880.33
	38025654.19	94727999.14	111413707.84	91213045.37
F/R	0.014843979	0.052682645	0.009326479	0.309859449
Efficiency index	3.549091952		33.22362528	

F/R =  average Firefly luciferase readout/ average Renilla luciferase readout.

Efficiency index =  TALEN (F/R)/ control (F/R).

### The repair of DSB elicited size modification of the adjacent (TG)_n_

To determine a appropriate dosage, different doses of the DSTR TALEN mRNA pair were microinjected into 1-cell stage zebrafish embryos separatly. At the dosage of 125 pg per embryo, the injected embryos developed as normally as the uninjected control and exhibited no specific abnormality at 1 day post fertilization (Figure 2). When the dosage was increased to 250 pg per embryo, slightly higher percentage of dead and unspecific deformed was observed (Figure 2), suggesting that substantial off-targeting was induced in this dosage. Therefore, the dosage of 125 pg per embryo was used in further experiment.

**Figure 3:**
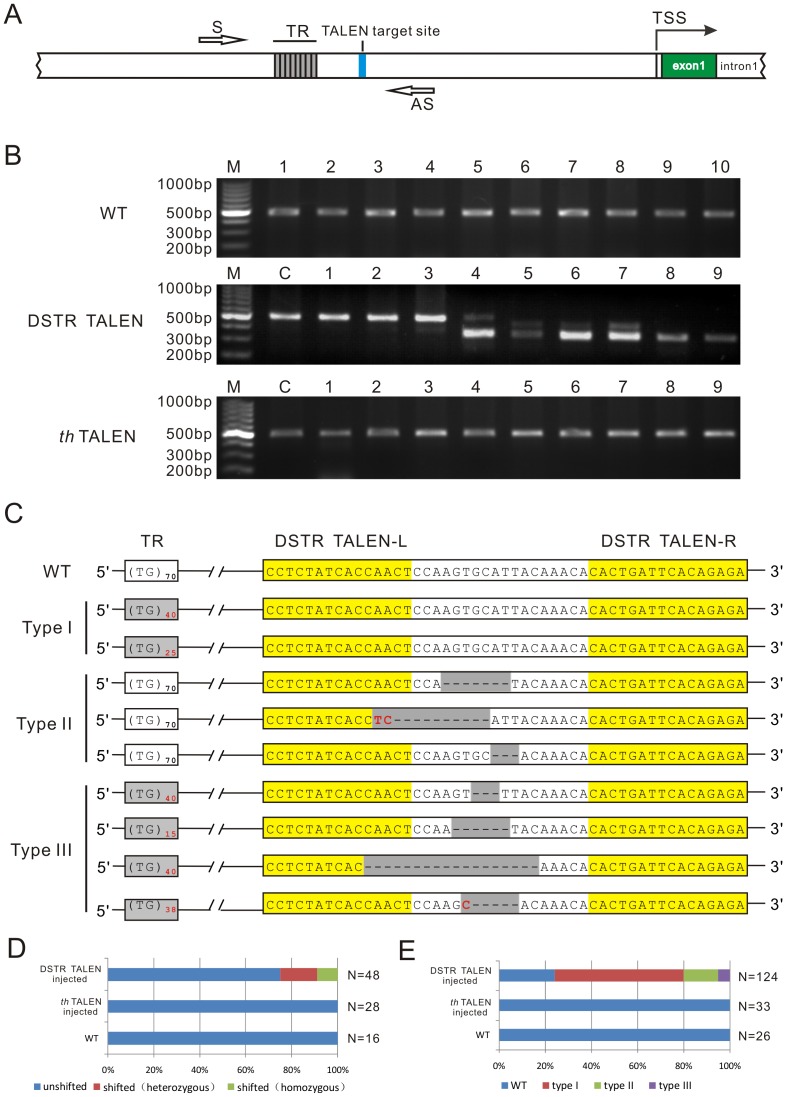
Mutants screening and classification in TALENs injected zebrafish embryos. (A) Analyzed region for screening potential mutant within the upstream sequence of zebrafish *ntl*. The tandem repeat (TR) is represented with grey adjacent boxes and the TALEN target site is shown in blue box. Primer pair used for PCR amplification of the potential mutant region (S and AS for sense and anti-sense primers, respectively) is indicated in blank arrows. (B) Agarose gel electrophoretogram of PCR products from wild type (WT), *th* TALEN and DSTR TALEN injected individuals. The DSTR TALEN samples are the selected representative individuals being sequencing analyzed. M. markers, C. wild type control. (C) Sequencing analysis and classification of mutants. Various mutations can be divided into three types. The number of TG unit (N) in wild type (WT) is about 70. No more than 3 TG unit of variation was detected in the strain used in this study. Significant size changes in the mutants were highlighted in red number. The changes of different indels in the sequence of the TALEN target site were highlighted in grey. Deletions and insertions were indicated by dashes and red letters, respectively. The binding sites of left and right TALENs (DSTR TALEN-L and TALEN-R) are highlighted in yellow. (D) Percentages of samples with unshifted and shifted (heterozygous and homozygous) bands detected by PCR amplification. (E) Percentages of each mutation types in TALEN-injected embryos. Only the sequenced clones in the shifted heterozygote and homozygote were calculated.

To sreen and analyze mutants, a ∼470bp fragment encompassing the TR and the DSRT TALEN target site upstream of *ntl* was PCR amplified (Figure 3A) from genomic DNA of single zebrafish embryos at 4 days post fertilization. In all examined wild type individuals (N = 16), the amplified fragment is almost the same. However, about 27.1% of the examined DSTR TALEN injected embryos (N = 48) exhibited obvious shifted bands in heterozygotic or homozygotic manner (Figure 3B, D). To confirm that the length change at the TR locus was specifically trigggered by DSTR TALEN, rather than by the injection or subsequent handing of the embryos, a published *th* TALEN pair targeting the *tyrosine hydroxylase* gene [25] was emploied a control. In all the examined control *th* TALEN embryos (N = 28), the length of the amplified fragment is the same as observed in wild type embryos (Figure 3B, D). These results indicated that the change in size of the fragment was unequivocally induced by DSTR TALEN.

The sequence of PCR products amplified from representative individuals were analyzed. To detect different clones in an single embryo, more than 10 clones from each of the individuals were sequenced and analyzed. Sequencing analysis of all the examined clones (N = 124) obtained from the heterozygotic or homozygotic mutant embryos showed that there were three types of mutation pattern (Figure 3C, E ). About 56.3% of the sequenced clones was classified into type I. In this type the (TG)_n_ was variably shortened, while the sequence of the TALEN target site remained unchanged. About 14.5% of the sequenced clones was classified into type II. In this type the (TG)_n_ remained unchanged, while various indels emerged at the TALEN target site. About 5% of the sequenced clones was classified into type III. In this type sequence change occurred within both the (TG)_n_ and the TALEN target site. Most of the examined embryos are heterozygotes or mosaics containing two or three types of clones (Table 2). Strikingly, the TR was contracted to various sizes and the shortest one maintained only 21% of the repeat unit. These results demonstrated that the TALEN-induced DSB resulted in mutations not only at the DSB site but also within the adjacent TR region.

**Table 2:** Type of clones in the examined DSTR TALEN individuals.

No. of samples	Type of clones			
1	Wt			
2	Wt		Type II	
3	Wt		Type II	
4	Wt	Type I	Type II	
5		Type I	Type II	Type III
6		Type I		Type III
7		Type I	Type II	
8		Type I		
9		Type I		

## Discussion

Since its discovery several years before, TAL effector fused with *Fok* I nuclease has been widely applied in targeting genes in plant [26], nematodes [27], fishes [28] and mammals [29], [30]. In this experiment, we successfully modified the size of a tandem repeat (TG)_n_ in zebrafish genome using engineered TALENs. Our results also showed that, during the repair of the DSB induced by TALENs, mutations can occur separately within the TALEN target region or within the adjecent TR region, or simutaniusly at both regions. Therefore, all the desired mutant types of TR could be convenintly obtained by employing enginered TALENs.

The tandem repeat (TG)_n_ was first discovered in the genomes from yeast to human in early 1980s by two independent groups [31], [32]. The following *in vitro* experiments showed that the activity of *chloramphenicol acetyltransferase* gene was enhanced with a TG-element, and the maximum enhancement was obtained with 30–40bp of (TG)_n_; when the (TG)_n_ exceeded 130bp, the gene activity declined dramatically to fivefold less compared with a 50bp (TG)_n_ [33]. Dutreix et al also showed *in vitro* that the binding of RecA protein to (TG)_ n_ or (CA)_ n_ sequences with an increasing affinity, and the sequence recombination was promoted from 30% to 80% and 100% for DNA containing 7, 16 and 39 TG repeats [34]. These observations suggested that (TG)_n_ played an important role in modulating gene expression. Recent publications suggest that the loss or gain of repeats may affect the binding of the transcriptional regulatory proteins such as IHF to the promoter [14], and that variations in repeats length can also affect gene expression through changing local nucleosome positioning and chromatin structure [15]. It is also proposed that repeats length variations might also affect DNA methylation [35]–[37]. The modification of the size of (TG)_n_ region and its flanking sequence *in vivo* provides a strategy to explore the detailed mechanisms and the roles of (TG)_n_ in gene expression and DNA methylation.

**Figure 4:**
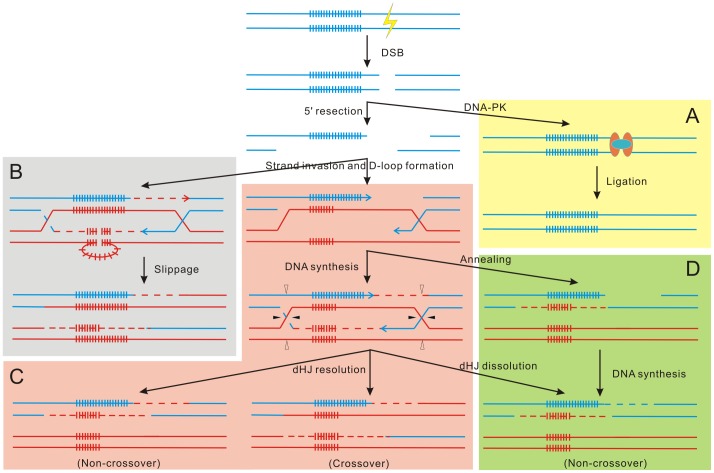
Speculative mechanisms involved in TALEN induced DSB repair. Blue lines represent the genome with DSB sites, and clusters of vertical bars indicate the TR region. The DSB ends can be bound by two groups of proteins independently: the binding of DNA-dependent protein kinase (DNA-PK) complex (Ku 70 and Ku80) and the following ligase IV seals the gap by direct rejoin the broken ends, which is termed non-homologous end-joining (NHEJ) pathway (A); While the binding of MRN complex and Exo1 nuclease initiates the 5'-3' resection of the ends, which is followed by either a replication slippage pathway (B) or homologous recombination (HR) pathway (C, D). In the replication slippage pathway, the TR region forms a secondary structure and leads to mispairing between the template and the newly-synthesized DNA strand. In the HR pathway, the 3' overhang invades into the homologous template DNA (red lines) and primes DNA synthesis (dash lines) to form a structure called D-loop, which will result in a double Holliday junction (dHJ). dHJ can either be resolved by strand cleavage with or without crossover, which is referred as classical DSB repair (DSBR) pathway of HR (C), and dHJ can also be dissolved by helicases to generate a non-crossover (D). Alternatively, D-loop can be directly dissociated through a synthesis-dependent strand annealing (SDSA) pathway, which results in exclusively non-crossover products (D).

Although several hypothesis, including replication slippage [38], gene conversion [39], and unequal crossing over [40] have been proposed, the actual molecular mechanisms involved in TRs instability remains unproved. In this experiment, the engineered TALEN pair induced a DSB adjacent to the (TG)_n_ region in zebrafish genome and elicited three types of mutations with or without TR variation. The type II mutation with indels within the TALEN target region while the (TG)_n_ region unchanged is undoubtedly due to non-homologous end-joining (NHEJ, Figure 4A), which is an error-prone pathway and hence usually introduce in mutations. Protein factors involved in this progress are well characterized, including DNA-dependent protein kinase (DNA-PK) complex (Ku 70 and Ku80) and ligase IV, which have been extensively reviewed recently [41]. The type I and type III mutation with repeat number variation in the (TG)_n_ region might be caused by replication slippage and homologous recombination (HR) through several pathways as illustrated in Figure 4B-D. Replication slippage is also called slipped-strand mispairing, in which the TR region forms a secondary structure and leads to mispairing between the template and the newly-synthesized DNA strand. The TR will contract, as observed in this experiment, if the template strand loops out (Figure 4B) and will expand if the newly-synthesized strand loops out. HR is a template-dependent repair progress and requires the formation of a displacement-loop (D-loop) followed by a DNA cross structure called double Holliday junction (dHJ), which can be resolved by strand cleavage with or without crossover and also can be dissolved by helicases to generate a non-crossover (Figure 4C and D). Alternatively, D-loop can also be dissociated through a synthesis-dependent strand annealing (SDSA) pathway, which results in exclusively non-crossover products (Figure 4D). The choice between NHEJ and HR after DSB might be dependent on the species, cell type and stage of the cell cycle [42], [43]. On the molecular level, the binding of Ku70-Ku80 heterodimer to DSB site and the following recruiting of ligase IV prevent the 5' resection and the following HR progress, while the 5'-3' resection initiated by MRN complex and Exo1 nuclease greatly antagonizes the NHEJ pathway [44]. Former studies showed that DSBs elicit the TR instability on a number of occasions [45]–[47]. Recent studies reported that the probability of HR is greatly enhanced when high doses of donor template sequences are supplied [48], [49]. It is possible that co-injection of engineered TALEN mRNAs with corresponding donor templates will gain precisely desired length of TRs.

## Supporting Information

File S1 **Combined file of supporting information files.** The contents include: Table S1. Primers for goldfish *ntl* promoter cloning. Table S2. Primers for pSSA-luciferase reporter construction. Figure S1. Alignment of upstream sequence of *ntl* between bisexual diploid and unisexual polyploid goldfish. Figure S2. Simplified structure of pSSA-luciferase reporter and sketch map of the SSA assay.
(DOC)
