# Supplementary material for: Tandem Repeat Modification during Double-Strand Break Repair Induced by an Engineered TAL Effector Nuclease in Zebrafish Genome
Source: PLoS One. 2013 Dec 26;8(12):e84176. doi: 10.1371/journal.pone.0084176 (PMC3873399; doi:10.1371/journal.pone.0084176)
Supplement: File S1 — Combined file of supporting information files. The contents include: Table S1. Primers for goldfish ntl promoter cloning. Table S2. Primers for pSSA-luciferase reporter construction. Figure S1. Alignment of upstream sequence of ntl between bisexual diploid and unisexual polyploid goldfish. Figure S2. Simplified structure of pSSA-luciferase reporter and sketch map of the SSA assay. (DOC) [file pone.0084176.s001.doc]

**Supplementary Materials**

**Tandem repeat modification during double-strand break repair induced by an engineered TAL effector nuclease in zebrafish genome**

Wanxu Huang^#^, Jianbo Zheng^#^, Ying He, Chen Luo*

College of Life Sciences, Zhejiang University, Hangzhou, Zhejiang 310058, China

# These authors contributed equally to this work.

* Corresponding author: Chen Luo; Email: luoc@zju.edu.cn

**Table S1. Primers for goldfish *ntl* promoter cloning**

| **Primer name** | **Sequence (5'-3')** | **Tm (°C)** |
| --- | --- | --- |
| GF-*ntl*-GSP5 | AGAATGATGATGGAGGCTGATG | 59.0 |
| GF-*ntl*-GSP6 | GTTTTACGAGGGCTCTGTGAATC | 60.0 |
| GF-*ntl*-GSP7 | TCTGTATTTTGCATGCGTGCTGACTGTG | 71.2 |
| GF-*ntl*-GSP8 | AGTGCTGGAGGGGTTAAGAGGAGATGCT | 70.9 |
| GF-ntl-promt-seq-S | CTTATGGTCAGTAATAATTTATTTC | 52.8 |
| GF-ntl-promt-seq-AS | TTAGTGACAATCATTTCATTAGTG | 52.5 |

**Table S2. Primers for pSSA-luciferase reporter construction**

| primer name | sequence (5'-3') | RE site |  |
| --- | --- | --- | --- |
|  | ZF-*ntl*-TALEN1-SSA-F | TATGTGAAGATCTATTCAGTTTATTCCCTGCTTT | *Bgl* II |
|  | ZF-*ntl*-TALEN1-SSA-R | ATGTCGCTCGAGTTATTAACTCAACATCCATGC | *Xho* I |
|  | ZF-*ntl*-TALEN2-SSA-F | TATGTGAAGATCTTGACAGGCTGTTTGTGAATAT | *Bgl* II |
|  | ZF-*ntl-*TALEN2-SSA-R | AATGTCGCTCGAGTGTGGAGTGTTTGAAGTTGAA | *Xho* I |

RE represents restriction endonuclease.

The recognizing site of RE is underlined in the primer sequence.


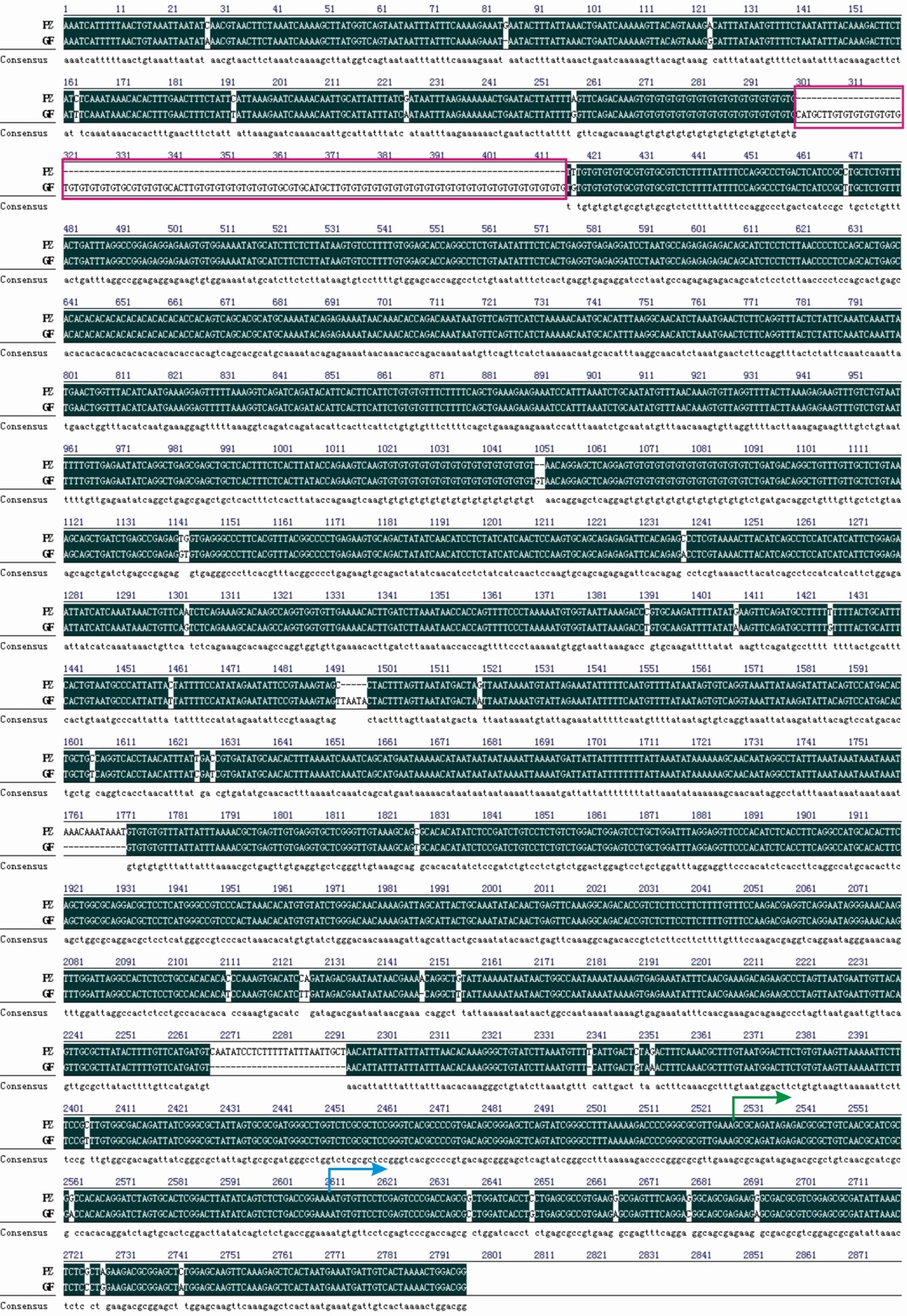


**Figure S1. Alignment of upstream sequence of *ntl* between bisexual diploid and unisexual polyploid goldfish.** GF: bisexual diploid goldfish (*Carassius auratus*), PZ: unisexual polyploid goldfish (*Carassius auratus*, pengze). Green and blue arrows indicate transcription and translation start site, respectively. The indel of an imperfect tandem repeat is boxed.


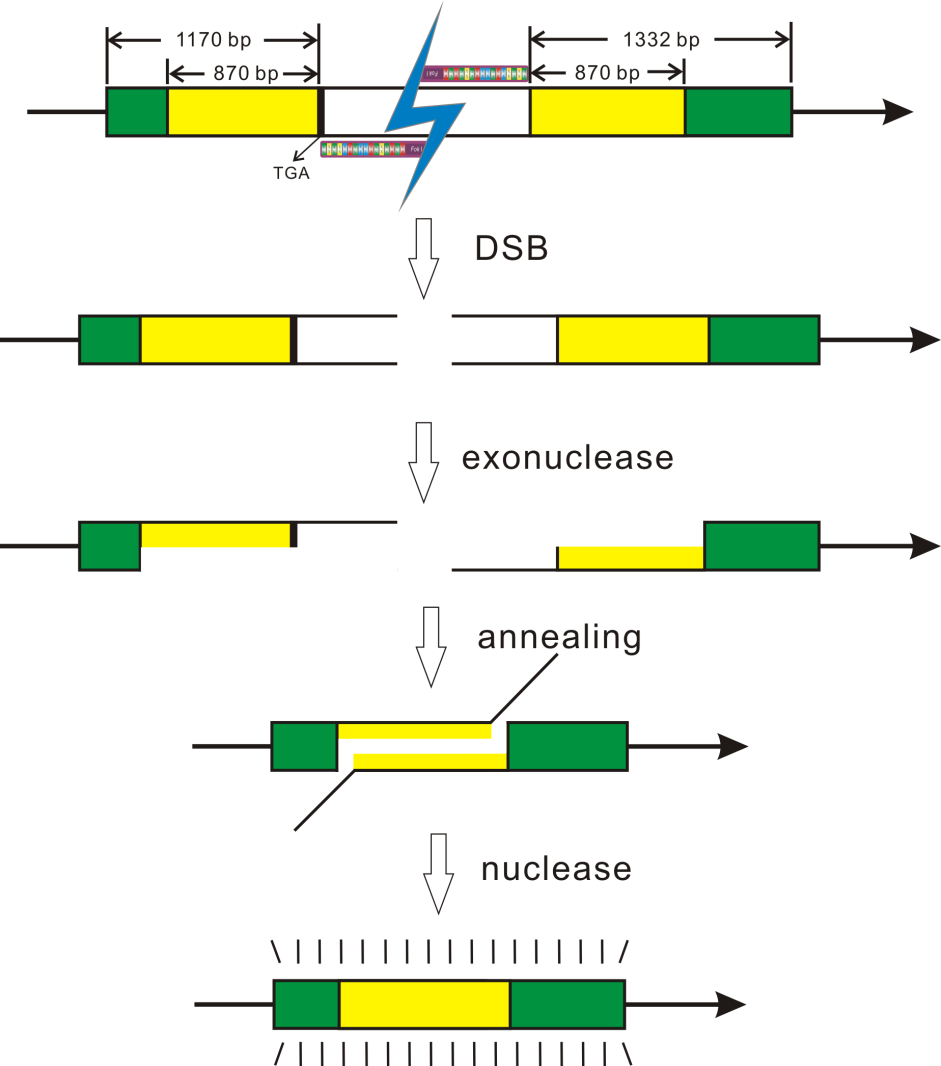


**Figure S2.** **Simplified structure of pSSA-luciferase reporter and sketch map of the SSA assay.** The reporter is driven by a CMV promoter. The green and yellow boxes indicate the coding region of the reporter firefly luciferase which is engineered with two 870bp homologous arms (yellow box) separated by a stop codon (TGA) and TALEN target sequence (white box). The binding of a TALEN pair creates a double strand break (DSB) at the target, which will soon be repaired through a single strand annealing (SSA) recombination pathway, in which the DNA strand is resected by exonuclease such as MRE11 to leave single strand DNA (ssDNA) overhangs [49], and after annealing of the two homologies on the exposed strands, the remaining overhangs are trimmed and eventually results in an active firefly luciferase gene.
